# Complicated Diverticulitis With Associated Small Bowel Obstruction: A Case Report

**DOI:** 10.7759/cureus.28942

**Published:** 2022-09-08

**Authors:** Florian A Walter, Gilbertas Rimkus

**Affiliations:** 1 Transitional Year Residency, Campbell University/Conway Medical Center, Conway, USA; 2 General Surgery, Campbell University/Conway Medical Center, Conway, USA

**Keywords:** ileostomy closure, surgical anastomosis, diverticular bowel obstruction, small-bowel obstruction, sigmoid diverticulitis

## Abstract

Diverticulitis is a common disease of the colon with a broad spectrum of presentations. This case outlines a less commonly seen presentation of delayed complicated diverticulitis with abscess formation and associated small bowel obstruction.

This report outlines the case of a 58-year-old obese male who initially presented to the emergency department with uncomplicated diverticulitis, was discharged home, and returned two days later with worsening symptoms. A repeat computed tomography (CT) scan demonstrated abscess formation with an associated small bowel obstruction. He underwent midline exploratory laparotomy with adhesiolysis of the portion of obstructed small bowel and resection of the diseased segment with primary anastomosis and diverting loop ileostomy. He had an ileostomy reversal at a later date.

Small bowel obstruction is a less observed complication of diverticulitis. This report serves to educate on the presentation of these patients as well as to discuss the potential surgical management of these patients. If this patient had presented without the small bowel obstruction, he may have just needed percutaneous drainage.

## Introduction

Acute colonic diverticulitis is inflammation of the colonic diverticula, most commonly caused by occlusion of the colonic diverticula leading to bacterial overgrowth and inflammation. The typical presentation is acute onset of lower abdominal pain, classically in the left lower quadrant, with systemic signs and symptoms, including nausea, vomiting, fever, and chills. Risk factors include obesity, a sedentary lifestyle, and the use of non-steroidal anti-inflammatory medications [1]. It is a common gastrointestinal disease, accounting for approximately 1.9 million outpatient visits and 208,000 inpatient admissions in the United States [2]. The incidence is estimated at 180/10,000 persons per year [3].

The disease can be further classified into uncomplicated or complicated, determined largely based on a computed tomography (CT) scan of the abdomen and pelvis. The uncomplicated disease is characterized by colonic wall thickening and peri-colonic inflammatory changes. Complicated diverticulitis includes colonic perforation, colonic stricture, colonic obstruction, and the formation of abscesses and fistulas [2]. A majority of patients presenting with uncomplicated diverticulitis can be appropriately treated outpatient with or without oral antibiotic therapy [1].

Approximately 12% of cases of uncomplicated diverticulitis will progress to complicated diverticulitis. The most common presentation of complicated diverticulitis is the formation of an abscess or phlegmon [2]. Diverticular abscesses measuring greater than 4 centimeters often require the placement of a percutaneous drain with elective resection of the affected colon at a later date. However, 15-30% of patients admitted to the hospital with acute diverticulitis require surgical intervention [1]. This case report describes a patient who initially presented with uncomplicated diverticulitis and then progressed to complicated diverticulitis with abscess formation, leading to small bowel obstruction.

## Case presentation

**Figure 1:**
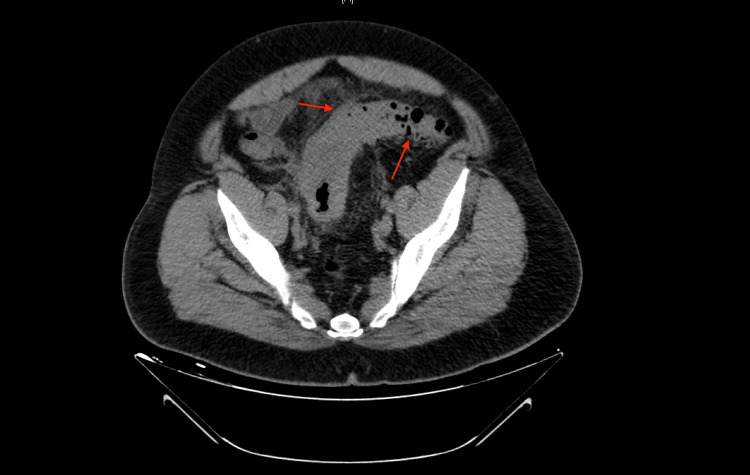
Transverse section of CT abdomen and pelvis without contrast from the initial emergency department visit showing colonic wall thickening and fat stranding (red arrows) consistent with uncomplicated diverticulitis

This patient is a 58-year-old obese male with a history of type II diabetes mellitus and prior uncomplicated diverticulitis two years ago, who initially presented to the emergency department with one week of constant left lower quadrant abdominal pain. Laboratory work and CT of the abdomen and pelvis were obtained at that time. Labs were remarkable for leukocytosis and a CT scan of the abdomen and pelvis without contrast was obtained, demonstrating uncomplicated diverticulitis as shown in Figure 1. Per the patient's history, he has never had a colonoscopy. He was discharged home on an oral antibiotic regimen of ciprofloxacin and metronidazole and with outpatient follow-up.

**Figure 2:**
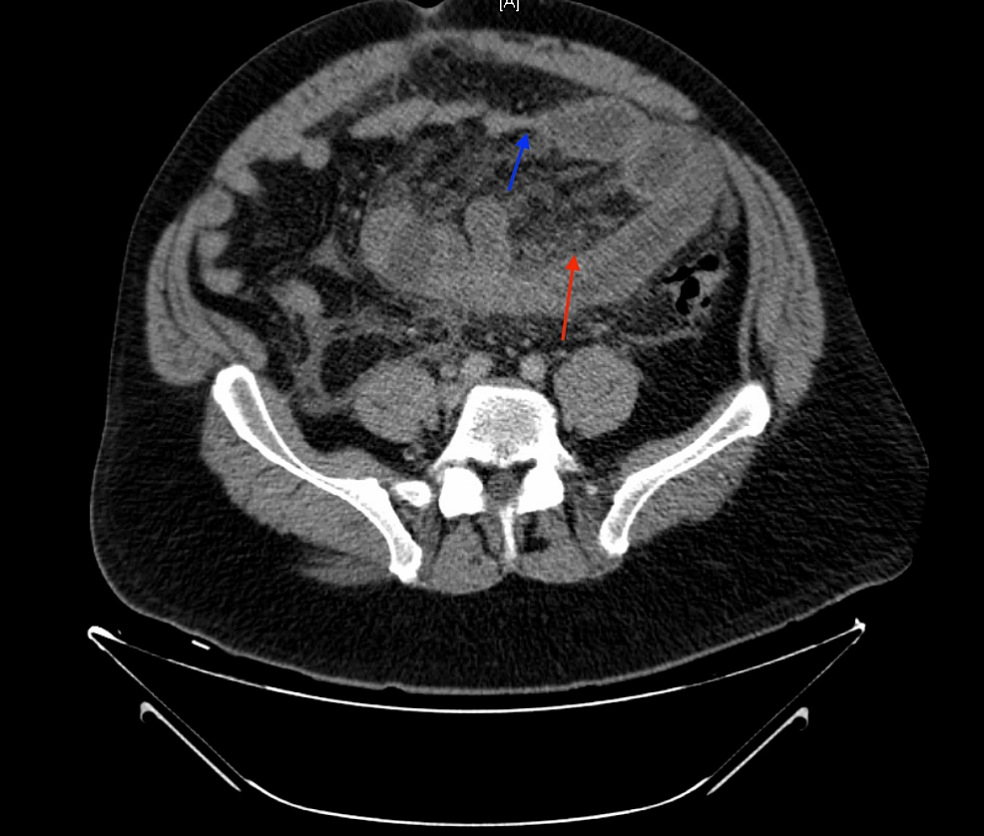
Transverse section of CT abdomen and pelvis with IV contrast from the subsequent emergency department visit, demonstrating inflammatory changes consistent with diverticulitis (red arrow) and distal small bowel decompression with proximal small bowel dilation (blue arrow) consistent with small bowel obstruction

**Figure 3:**
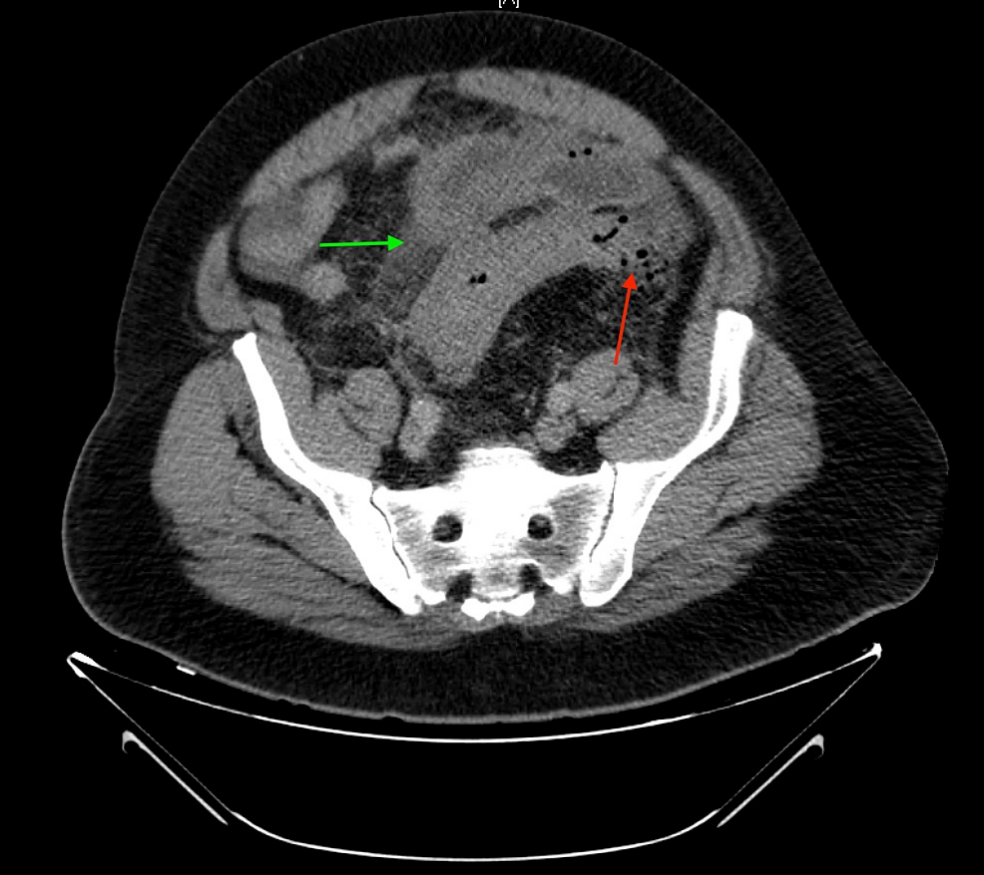
Transverse section of CT abdomen and pelvis with IV contrast from the subsequent emergency department visit, demonstrating sigmoid colonic wall thickening, fat stranding consistent with diverticulitis (red arrow), and the development of an encapsulated fluid collection consistent with abscess formation (green arrow)

He returned to the emergency department two days later with worsening left lower quadrant pain. Repeat blood work and a second CT of the abdomen and pelvis with intravenous (IV) contrast were obtained as shown in Figure 2 and Figure 3. Laboratory testing demonstrated an increasing white blood cell count at 23,000 K/mm3. CT demonstrated a diverticular abscess formation associated with a small bowel obstruction with a transition point near the abscess. A nasogastric tube was placed, IV fluids were started, and the patient was started on an IV antibiotic regimen of ertapenem. He was admitted to the surgery service for further management.

Informed written consent was obtained from the patient, and he was taken to the operating room for exploratory laparotomy. Intraoperatively, the inflammatory mass was found in the sigmoid colon with a portion of the small bowel attached. The small bowel adhesions were carefully dissected from the mass, and the inflamed portion of the sigmoid colon was resected. A side-to-side anastomosis was created, utilizing a gastrointestinal anastomosis stapler and reinforced with silk sutures. On inspection of the anastomosis, there appeared to be some narrowing in the proximal colon and a diverting loop ileostomy to protect the anastomosis. The fascia was reapproximated and the skin was closed loosely with staples.

On postoperative day one, he was tolerating his diet and ambulation. The ileostomy was productive of flatus and feculent material. He remained on ertapenem and his white blood cell count was monitored for improvement until his discharge on postoperative day seven. He returned to the outpatient clinic two weeks after his discharge from the hospital and was progressing appropriately. A barium enema was ordered to evaluate the anastomosis prior to scheduling ileostomy reversal. The barium enema had no evidence of anastomotic leak or stricture. Ileostomy reversal was performed six weeks after the initial operation. He was monitored in the hospital for three days after the ileostomy reversal and discharged home after the return of normal bowel function. He will have regular follow-ups in the general surgery clinic and be monitored for any potential complications.

## Discussion

This presentation of colonic diverticulitis with associated small bowel obstruction is an uncommon presentation of this common disease, and CT evaluation of these complications is previously described by Kim et al. [4]. Colonic diverticulitis accounts for 10-15% of cases of colonic obstruction, however, it is a less established cause of isolated small bowel obstruction [4]. Approximately 1.4-4.3% of hospitalized patients with complicated diverticulitis will need emergent surgery during their admission [5].

There is a sparse discussion of the frequency or treatment of this complication in recent literature. However, four similar cases are described by Welch and Warsaw in 1977 [6]. In these cases, acute diverticulitis was masked by the small bowel obstruction. Anatomic proximity of the sigmoid colon to the small bowel allows the small bowel to become entrapped in the inflammatory process caused by acute diverticulitis, leading to a mechanical small bowel obstruction. Feared complications of mechanical small bowel obstruction include small bowel necrosis or small bowel perforation [7].

This case demonstrates another example of this presentation while also describing the surgical management and postoperative course. The patient had risk factors for developing diverticulitis, including obesity and a sedentary lifestyle [3]. He did not respond to the initial outpatient oral antibiotic regimen, and his disease course progressed rapidly. Standard treatment of complicated diverticulitis with abscess formation can be treated conservatively with antibiotics and possibly percutaneous drainage [5]. The decision for percutaneous drainage is made based on the radiographically measured size of the abscess. Typically, abscesses greater than 4 centimeters are considered for percutaneous drainage or surgical intervention [5]. Without the small bowel obstruction, this patient would have met the criteria for percutaneous drainage of his abscess. However, due to the risks associated with small bowel obstruction and rapidly worsening clinical status, the decision was made to operate on this patient.

## Conclusions

The purpose of this report is to substantiate the spectrum of severity of diverticulitis and the possible complication of associated small bowel obstruction secondary to abscess formation. This is an uncommon presentation that should be considered in patients with signs of small bowel obstruction with diverticular abscess formation. This case describes a potential strategy for the surgical management of these patients. Future studies could be done to evaluate the frequency and management of this complication in clinical practice.
